# Two Cases of Rupture of the Uterus

**Published:** 1896-08

**Authors:** M. Giovanolli


Two Cases of Rupture of the Uterus. By M. Giovanolli. Circumscribed lesions of the matrix which do not penetrate deeply are rather frequent in the cow and often pass unnoticed; rarely they cause an infectious metritis. These lesions are produced especially during delivery; they are met with, however, before the fœtus has arrived in the vagina. The author was called in to a cow which had passed the waters several hours previous, and which did not seem to be suffering severe pain; she looked at her abdomen from time to time and moved her tail. On investigating with the hand, M. Giovanolli affirmed that the mouth of the uterus was well opened and that the nose of the calf and two of the legs had entered it; he asserted that everything presented normal conditions. The examination had caused several violent expulsive efforts on the part of the animal. At the end of ten minutes the author again introduced his hand into the genital passages; but, to his great astonishment, he could no longer feel the calf. On the contrary, he found a transverse rent on the lower wall of the uterus through which the fœtus had passed. The animal was killed, when a large laceration was found situated about five centimetres from the neck of the uterus; toward the middle the edges of the rent were cicatrized, but elsewhere they were bleeding. M. Giovanolli thinks that a small laceration existed previously, and that it was enlarged by the expulsive efforts. In a second case the subject was a cow two months overdue. On examination the author drew out a small fœtus accompanied by envelopes; wishing to disinfect the matrix, immediately he discovered the existence of a laceration on the upper surface about four fingers long; the matrix was completely empty. On account of the position of the laceration he advised the owner to preserve the animal, which recovered, became pregnant again and was delivered normally the following year. The author thinks that in consequence of the weight of the fœtus a sort of shock or violent tension is produced at the level of the neck at the moment of uterine contractions; this shock could cause the wall to become thinner, and under the influence of special mechanical conditions and violent contractions a rent more or less extensive is produced.—From the *Schweizer Archiv* in the *Annales de Méd. Vétérinaire*, for Jan. and Feb., 1896.

